# Psychopathological networks in psychosis and changes over time: A long-term cohort study of first-episode psychosis

**DOI:** 10.1192/j.eurpsy.2022.637

**Published:** 2022-09-01

**Authors:** G. Gil-Berrozpe, V. Peralta, A. Sánchez-Torres, L. Moreno-Izco, E. Garcia De Jalon, D. Peralta, L. Janda, M. Cuesta

**Affiliations:** Instituto de investigación Sanitaria de Navarra (IdiSNA), Psychiatry Unit, Complejo Hospitalario De Navarra, Pamplona, Spain

**Keywords:** Network Analysis, First-episode psychosis, Long-term, Psychopathology

## Abstract

**Introduction:**

First-episode psychosis is a critical period for early interventions to reduce the risk of poor outcomes and relapse as much as possible. There are now many studies revealing the patterns of course in the short and medium terms, but uncertainties about the long-term outcomes of symptomatology remain to be ascertained.

**Objectives:**

First, we ascertained whether the structure of psychopathological symptoms, dimensions and domains of psychopathology remains invariant over time between first-episode psychosis and long-term follow-up. Second, we analysed the changes in the interrelationships of psychopathological symptoms, dimensions and domains of psychopathology between FEP and long-term follow-up at three levels.

**Methods:**

We performed network analysis to investigate first-episode and long-term stages of psychosis at three levels of analysis: micro, meso and macro. The sample was a cohort of 510 patients with first-episode psychoses from the SEGPEP study, who were reassessed at the long-term follow-up (n = 243). We used the Comprehensive Assessment of Symptoms and History (CASH) for their assessments.

**Results:**

Our results showed a similar pattern of clustering between first episodes and long-term follow-up in seven psychopathological dimensions at the micro level, 3 and 4 dimensions at the meso level, and one at the macro level. They also revealed significant differences between first-episode and long-term network structure and centrality measures at the three levels.

**Conclusions:**

Our findings suggest that disorganization symptoms have more influence in long-term stabilized patients. The main results of the current study add evidence to the hierarchical, dimensional and longitudinal structuring of first-episode psychoses.

**Disclosure:**

No significant relationships.

